# Bis[bis­(diphenyl­thio­phosphin­yl)amido-κ^2^ *S*,*S*′]platinum(II)

**DOI:** 10.1107/S1600536811010117

**Published:** 2011-03-26

**Authors:** Cemal Güzelsoylu, Sevil Irişli, Orhan Büyükgüngör

**Affiliations:** a University of Ege, Faculty of Science, Department of Chemistry, 35100 Izmir, Turkey; b University of Ondokuzmay, Faculty of Science, Department of Physics, 55139 Samsun, Turkey

## Abstract

In the title compound, [Pt(C_24_H_20_NP_2_S_2_)_2_], the Pt atom is in a distorted square-planar environment and contains two six-membered carbon-free chelate rings, one in twist-boat and the other in a half-chair conformation. Two phenyl groups are disordered over two set of sites in ratios of 0.721 (13):0.279 (13) and 0.71 (7):0.29 (7).

## Related literature

For general background to imidodiphosphinedichalcogenides, see: Schmidpeter & Groger (1966 ▶); Woollins (1996 ▶); Haiduc (1997 ▶); Silvestru *et al.* (1998 ▶); Sekar & Ibers (2006 ▶); Crouch *et al.* (2003 ▶); Abbati *et al.* (2001 ▶). For related structures, see: Yanar *et al.* (2007 ▶); Bhattacharyya & Woollins (1995 ▶); İrişli & Yanar (2006 ▶); Berry *et al.* (1988 ▶). For geometric analysis, see: Cremer & Pople (1975 ▶)
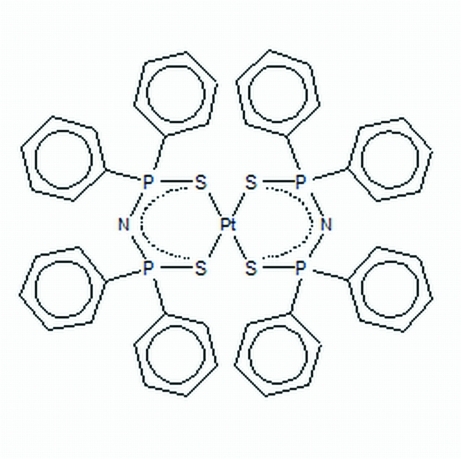


## Experimental

### 

#### Crystal data

[Pt(C_24_H_20_NP_2_S_2_)_2_]
*M* *_r_* = 1092.03
Triclinic, 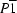

*a* = 10.1103 (4) Å
*b* = 10.7023 (4) Å
*c* = 23.9258 (9) Å
α = 98.137 (3)°
β = 90.496 (3)°
γ = 115.563 (3)°
*V* = 2304.86 (15) Å^3^
*Z* = 2
Mo *K*α radiation
μ = 3.40 mm^−1^
*T* = 296 K
0.56 × 0.32 × 0.08 mm

#### Data collection

Stoe IPDS 2 diffractometer
Absorption correction: integration (*X-RED32*; Stoe & Cie, 2002 ▶) *T*_min_ = 0.302, *T*_max_ = 0.790
23061 measured reflections
9495 independent reflections
8823 reflections with *I* > 2σ(*I*)
*R*_int_ = 0.033

#### Refinement

*R*[*F* ^2^ > 2σ(*F* ^2^)] = 0.026
*wR*(*F* ^2^) = 0.066
*S* = 1.03
9495 reflections
570 parameters
24 restraints
H-atom parameters constrained
Δρ_max_ = 0.82 e Å^−3^
Δρ_min_ = −0.79 e Å^−3^

### 

Data collection: *X-AREA* (Stoe & Cie, 2002 ▶); cell refinement: *X-AREA*; data reduction: *X-RED32* (Stoe & Cie, 2002 ▶); program(s) used to solve structure: *SHELXS97* (Sheldrick, 2008 ▶); program(s) used to refine structure: *SHELXL97* (Sheldrick, 2008 ▶); molecular graphics: *ORTEP-3 for Windows* (Farrugia, 1997 ▶); software used to prepare material for publication: *WinGX* (Farrugia, 1999 ▶).

## Supplementary Material

Crystal structure: contains datablocks I, global. DOI: 10.1107/S1600536811010117/jj2077sup1.cif

Structure factors: contains datablocks I. DOI: 10.1107/S1600536811010117/jj2077Isup2.hkl

Additional supplementary materials:  crystallographic information; 3D view; checkCIF report

## Figures and Tables

**Fig. 1.**
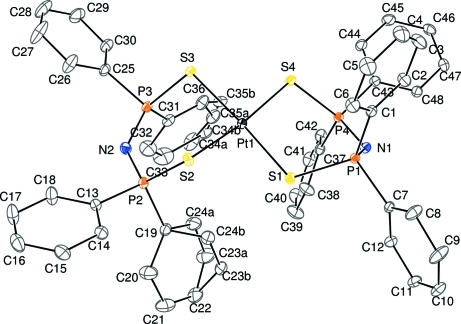
The perspective view of complex (I). Displacement ellipsoids are drawn at 20% probability level. H atoms have been omitted for clarity. Positional disorder occurs in the aromatic rings attached to the P2 and P3 atoms with the sof values of 0.721 (13) (C23A, C24A), 0.279 (13) (C23B, C24B), 0.71 (7) (C34A, C35A) and 0.29 (7) (C34B, C35B).

## supplementary crystallographic information

### Comment

In bis(diphenylphosphino)amine (dppa) ligands the phosphorous (III) atoms are succeptable to oxidation by chalcogene atoms (*i.e.* oxygene, sulfur and selenium). Further, double oxidation of dppa will generate bis(diphenylthiophosphoryl)-amine ligands [Ph_2_P(E)NHP(E)Ph_2_] (E= O, S, Se) and can act as a bidentate chelating anion (imidobis(diphenylthiophosphinato) with the loss of an amino proton to a metal metal ion (Schmidpeter & Groger, 1966). Similar compounds containing [*R*_2_P(E)N]_2_N^-^ (*R* = alkyl and phenyl) have been investigated (Woollins, 1996; Haiduc, 1997; Silvestru *et al.*, 1998). [*R*_2_P(E)N]_2_N^-^ ligands can also bonded to the metal atoms as a tridentate ligand through the two chalcogene and N atoms (Sekar & Ibers, 2006). Metal complexes of imidodiphosphino dichalcogenide ligands have been widely used as precursors for the thin films, nanoparticles (Crouch *et al.*, 2003). Iodine adducts of these ligands are used to activate the metals (Abbati *et al.*, 2001).

In the title complex, [Pt(Ph_2_P(S)N(S)Ph_2_)] or [Pt(dppaS_2_)_2_] where dppaS_2_ is bis[(imidobis(diphenylthiophosphinato)]platinum(II), the Pt atom is in a distorted square planar environment with angles *trans* S1—Pt1—S3 [172.37 (3)°] and S2—Pt1—S4 [171.72 (3)°] and S4—Pt1—S1 and S3—Pt1—S2 bite angles of 101.45 (3)° and 90.87 (3)°, respectively (Fig. 1). These values are comparable with literature values of related compounds [Yanar *et al.*, 2007; Bhattacharyya & Woollins, 1995]. The differences in the bite angles of S—Pt—S are likely due to the conformations of the six-membered metallo rings. In the Pt1/S1/P1/N1/P4/S4 ring, the Pt1—S1—P1—N1 and Pt1—S4—P4—N1 torsion angles are -57.12 (13)° and -51.12 (14)°, respectively, with ring puckering parameters of θ= 89.31 (11)° and Φ= 287.34 (13)°.  This supports a twist-boat conformation (Cremer & Pople,1975). In the Pt1/S2/P2/N2/P3/S3 ring with Pt1—S2—P2—N2 [-52.27 (16)°] and Pt1—S3—P3—N2 [51.49 (17)°] torsion angles and ring puckering parameters of θ= 180.52 (12)° and Φ= 141.81 (12)° (Cremer & Pople, 1975) supports a half-chair conformation. The four Pt—S bond lengths are slightly different from each other [Pt1—S1: 2.3284 (9) Å, Pt1—S2: 2.3207 (8) Å, Pt1—S3: 2.3300 (9) Å, Pt1—S4: 2.3341 (8) Å]. These values are comparable with literature values [Yanar *et al.*, 2007; İrişli & Yanar, 2006]. The observed P1—S1, P2—S2, P3—S3 and P4—S4 lengths [2.0279 (12) Å, 2.0332 (13) Å, 2.0327 (14)Å and 2.0304 (12) Å] are closer to single bond than double bond lengths (Berry *et al.*, 1988).

Positional disorder in the aromatic rings attached to the P2 and P3 atoms occur with sof values of 0.721 (13) (C23A, C24A), 0.279 (13) (C23B, C24B), 0.71 (7) (C34A, C35A) and 0.29 (7) (C34B, C35B), respectively.

### Experimental

As reactive substances, K_2_PtCl_4_ (0,108 g; 0.24 mmol) and dppaS_2_ (0.1 g; 0.24 mmol) were dissolved in dichloromethane-methanol solvent system, the stirring solution was refluxed overnight and then yellow solid was collapsed, washed with pentane and recrystalized from dichloromethane/diethylether. Complex (I) was also synthesized in the literature by a different reaction pathway (Bhattacharyya & Woollins, 1995).

### Refinement

All H atoms were refined using a riding model with C—H = 0.93 Å and *U*_iso_(H) = 1.2 *U*_eq_(C). There are positional disorders in the aromatic rings attached to the P2 and P3 atoms with the sof values of 0.721 (13) (C23A, C24A), 0.279 (13) (C23B, C24B), 0.71 (7) (C34A, C35A) and 0.29 (7) (C34B, C35B).

### Crystal data

**Table:**

[Pt(C_24_H_20_NP_2_S_2_)_2_]	*Z* = 2
*M**_r_* = 1092.03	*F*(000) = 1088
Triclinic, *P*1	*D*_x_ = 1.574 Mg m^−^^3^
Hall symbol: -P 1	Mo *K*α radiation, λ = 0.71073 Å
*a* = 10.1103 (4) Å	Cell parameters from 23061 reflections
*b* = 10.7023 (4) Å	θ = 1.7–27.3°
*c* = 23.9258 (9) Å	µ = 3.40 mm^−^^1^
α = 98.137 (3)°	*T* = 296 K
β = 90.496 (3)°	Prism, yellow
γ = 115.563 (3)°	0.56 × 0.32 × 0.08 mm
*V* = 2304.86 (15) Å^3^	

### Data collection

**Table:**

Stoe IPDS 2 diffractometer	9495 independent reflections
Radiation source: fine-focus sealed tube	8823 reflections with *I* > 2σ(*I*)
graphite	*R*_int_ = 0.033
rotation method scans	θ_max_ = 26.5°, θ_min_ = 1.7°
Absorption correction: integration (*X-RED32*; Stoe & Cie, 2002)	*h* = −12→12
*T*_min_ = 0.302, *T*_max_ = 0.790	*k* = −13→13
23061 measured reflections	*l* = −30→30

### Refinement

**Table:**

Refinement on *F*^2^	Primary atom site location: structure-invariant direct methods
Least-squares matrix: full	Secondary atom site location: difference Fourier map
*R*[*F*^2^ > 2σ(*F*^2^)] = 0.026	Hydrogen site location: inferred from neighbouring sites
*wR*(*F*^2^) = 0.066	H-atom parameters constrained
*S* = 1.03	*w* = 1/[σ^2^(*F*_o_^2^) + (0.036*P*)^2^ + 1.3319*P*] where *P* = (*F*_o_^2^ + 2*F*_c_^2^)/3
9495 reflections	(Δ/σ)_max_ = 0.002
570 parameters	Δρ_max_ = 0.82 e Å^−^^3^
24 restraints	Δρ_min_ = −0.79 e Å^−^^3^

### Special details

**Table:**

Geometry. All e.s.d.'s (except the e.s.d. in the dihedral angle between two l.s. planes) are estimated using the full covariance matrix. The cell e.s.d.'s are taken into account individually in the estimation of e.s.d.'s in distances, angles and torsion angles; correlations between e.s.d.'s in cell parameters are only used when they are defined by crystal symmetry. An approximate (isotropic) treatment of cell e.s.d.'s is used for estimating e.s.d.'s involving l.s. planes.
Refinement. Refinement of *F*^2^ against ALL reflections. The weighted *R*-factor *wR* and goodness of fit *S* are based on *F*^2^, conventional *R*-factors *R* are based on *F*, with *F* set to zero for negative *F*^2^. The threshold expression of *F*^2^ > σ(*F*^2^) is used only for calculating *R*-factors(gt) *etc*. and is not relevant to the choice of reflections for refinement. *R*-factors based on *F*^2^ are statistically about twice as large as those based on *F*, and *R*- factors based on ALL data will be even larger.

### Fractional atomic coordinates and isotropic or equivalent isotropic displacement parameters (Å^2^)

**Table:**

	*x*	*y*	*z*	*U*_iso_*/*U*_eq_	Occ. (<1)
C1	0.8948 (4)	0.3729 (3)	0.59754 (14)	0.0444 (7)	
C2	1.0207 (4)	0.4658 (4)	0.57643 (18)	0.0621 (9)	
H2	1.0238	0.5476	0.5661	0.074*	
C3	1.1416 (5)	0.4379 (6)	0.5706 (2)	0.0845 (14)	
H3	1.2266	0.5017	0.5574	0.101*	
C4	1.1354 (6)	0.3154 (6)	0.5844 (2)	0.0906 (16)	
H4	1.2162	0.2959	0.5800	0.109*	
C5	1.0115 (6)	0.2220 (6)	0.6044 (2)	0.0864 (15)	
H5	1.0082	0.1391	0.6135	0.104*	
C6	0.8916 (5)	0.2502 (4)	0.61133 (19)	0.0640 (10)	
H6	0.8079	0.1865	0.6253	0.077*	
C7	0.6165 (3)	0.3202 (3)	0.54114 (13)	0.0413 (6)	
C8	0.6247 (6)	0.2111 (5)	0.50558 (19)	0.0770 (13)	
H8	0.6995	0.1853	0.5123	0.092*	
C9	0.5221 (7)	0.1398 (6)	0.4599 (2)	0.0991 (19)	
H9	0.5282	0.0658	0.4363	0.119*	
C10	0.4126 (5)	0.1765 (4)	0.44895 (18)	0.0720 (12)	
H10	0.3434	0.1274	0.4184	0.086*	
C11	0.4052 (4)	0.2855 (4)	0.48304 (17)	0.0629 (10)	
H11	0.3317	0.3123	0.4753	0.076*	
C12	0.5062 (4)	0.3570 (4)	0.52921 (15)	0.0528 (8)	
H12	0.4992	0.4310	0.5525	0.063*	
C13	0.3674 (4)	0.1330 (4)	0.87188 (16)	0.0552 (8)	
C14	0.3001 (5)	0.0020 (5)	0.8382 (2)	0.0762 (13)	
H14	0.3096	−0.0051	0.7994	0.091*	
C15	0.2197 (6)	−0.1179 (5)	0.8606 (3)	0.0896 (15)	
H15	0.1756	−0.2050	0.8375	0.108*	
C16	0.2062 (6)	−0.1063 (6)	0.9175 (3)	0.0957 (17)	
H16	0.1498	−0.1861	0.9329	0.115*	
C17	0.2725 (8)	0.0178 (7)	0.9516 (3)	0.110 (2)	
H17	0.2637	0.0226	0.9904	0.132*	
C18	0.3546 (7)	0.1400 (5)	0.9293 (2)	0.0876 (15)	
H18	0.4004	0.2258	0.9533	0.105*	
C19	0.3316 (4)	0.3238 (4)	0.80632 (16)	0.0580 (9)	
C20	0.1798 (6)	0.2294 (6)	0.8039 (3)	0.1047 (19)	
H20	0.1489	0.1464	0.8188	0.126*	
C21	0.0774 (7)	0.2653 (8)	0.7783 (3)	0.120 (2)	
H21	−0.0224	0.2049	0.7758	0.144*	
C22	0.1238 (7)	0.3876 (7)	0.7573 (3)	0.0986 (17)	
H22	0.0576	0.4057	0.7366	0.118*	
C23A	0.2620 (12)	0.4801 (12)	0.7664 (6)	0.120 (4)	0.721 (13)
H23A	0.2900	0.5707	0.7589	0.144*	0.721 (13)
C24A	0.3672 (9)	0.4430 (10)	0.7871 (5)	0.097 (3)	0.721 (13)
H24A	0.4661	0.5046	0.7874	0.116*	0.721 (13)
C23B	0.245 (2)	0.397 (3)	0.7331 (9)	0.081 (6)	0.279 (13)
H23B	0.2656	0.4355	0.7000	0.097*	0.279 (13)
C24B	0.343 (2)	0.352 (3)	0.7540 (10)	0.089 (6)	0.279 (13)
H24B	0.4141	0.3421	0.7319	0.107*	0.279 (13)
C25	0.7966 (4)	0.5871 (4)	0.96474 (15)	0.0586 (9)	
C26	0.7835 (8)	0.4771 (6)	0.9929 (2)	0.107 (2)	
H26	0.7258	0.3844	0.9765	0.128*	
C27	0.8574 (12)	0.5080 (10)	1.0455 (3)	0.156 (4)	
H27	0.8484	0.4352	1.0646	0.187*	
C28	0.9427 (9)	0.6421 (10)	1.0699 (2)	0.127 (3)	
H28	0.9911	0.6606	1.1055	0.152*	
C29	0.9579 (7)	0.7498 (7)	1.0424 (2)	0.1047 (19)	
H29	1.0184	0.8418	1.0588	0.126*	
C30	0.8831 (5)	0.7222 (5)	0.99010 (19)	0.0776 (12)	
H30	0.8916	0.7962	0.9719	0.093*	
C31	0.6861 (5)	0.6984 (4)	0.88311 (17)	0.0619 (9)	
C32	0.5663 (7)	0.7108 (7)	0.9052 (3)	0.111 (2)	
H32	0.4996	0.6419	0.9238	0.133*	
C33	0.5481 (11)	0.8326 (10)	0.8989 (4)	0.148 (3)	
H33	0.4668	0.8403	0.9138	0.178*	
C34A	0.629 (4)	0.926 (2)	0.8758 (11)	0.112 (8)	0.71 (7)
H34A	0.6132	1.0050	0.8749	0.135*	0.71 (7)
C35A	0.743 (4)	0.914 (2)	0.8511 (7)	0.086 (6)	0.71 (7)
H35A	0.7993	0.9813	0.8298	0.103*	0.71 (7)
C34B	0.677 (5)	0.944 (5)	0.860 (2)	0.084 (11)	0.29 (7)
H34B	0.6607	1.0144	0.8473	0.101*	0.29 (7)
C35B	0.804 (6)	0.932 (4)	0.8483 (17)	0.071 (8)	0.29 (7)
H35B	0.8893	0.9999	0.8370	0.085*	0.29 (7)
C36	0.7814 (6)	0.8013 (5)	0.8561 (2)	0.0790 (13)	
H36	0.8657	0.7989	0.8418	0.095*	
C37	0.7337 (4)	0.7682 (3)	0.67965 (14)	0.0489 (7)	
C38	0.5841 (5)	0.6851 (5)	0.6757 (2)	0.0793 (13)	
H38	0.5474	0.5885	0.6648	0.095*	
C39	0.4883 (6)	0.7448 (6)	0.6880 (3)	0.1044 (19)	
H39	0.3875	0.6880	0.6845	0.125*	
C40	0.5400 (7)	0.8856 (6)	0.7053 (2)	0.0897 (15)	
H40	0.4752	0.9244	0.7150	0.108*	
C41	0.6860 (6)	0.9683 (5)	0.70833 (19)	0.0745 (12)	
H41	0.7211	1.0648	0.7190	0.089*	
C42	0.7838 (5)	0.9124 (4)	0.69590 (17)	0.0609 (9)	
H42	0.8841	0.9712	0.6984	0.073*	
C43	1.0174 (4)	0.8296 (3)	0.63714 (14)	0.0448 (7)	
C44	1.1319 (4)	0.9254 (4)	0.67540 (18)	0.0662 (11)	
H44	1.1286	0.9183	0.7137	0.079*	
C45	1.2513 (5)	1.0315 (5)	0.6567 (2)	0.0773 (13)	
H45	1.3279	1.0958	0.6825	0.093*	
C46	1.2566 (4)	1.0421 (4)	0.6001 (2)	0.0654 (10)	
H46	1.3376	1.1123	0.5875	0.078*	
C47	1.1428 (4)	0.9494 (4)	0.56251 (18)	0.0608 (9)	
H47	1.1460	0.9579	0.5243	0.073*	
C48	1.0225 (4)	0.8429 (3)	0.58052 (15)	0.0507 (8)	
H48	0.9453	0.7805	0.5546	0.061*	
N1	0.7810 (3)	0.5722 (2)	0.60647 (11)	0.0416 (5)	
N2	0.5590 (3)	0.4148 (3)	0.89129 (13)	0.0562 (7)	
P1	0.73768 (8)	0.41014 (7)	0.60417 (3)	0.03652 (15)	
P2	0.46930 (10)	0.28923 (9)	0.84166 (4)	0.04727 (18)	
P3	0.71250 (10)	0.54698 (9)	0.89377 (4)	0.04749 (19)	
P4	0.85814 (8)	0.69144 (7)	0.66001 (3)	0.03865 (16)	
S1	0.61827 (9)	0.31817 (8)	0.66701 (3)	0.04800 (18)	
S2	0.58863 (10)	0.23147 (8)	0.78461 (4)	0.04756 (18)	
S3	0.86728 (9)	0.52805 (9)	0.84426 (3)	0.04726 (17)	
S4	0.93207 (9)	0.63793 (8)	0.72772 (4)	0.04849 (18)	
Pt1	0.746243 (12)	0.437756 (11)	0.754094 (4)	0.03693 (4)	

### Atomic displacement parameters (Å^2^)

**Table:**

	*U*^11^	*U*^22^	*U*^33^	*U*^12^	*U*^13^	*U*^23^
C1	0.0485 (17)	0.0486 (16)	0.0385 (16)	0.0237 (14)	0.0012 (13)	0.0065 (12)
C2	0.060 (2)	0.065 (2)	0.071 (3)	0.0325 (18)	0.0171 (19)	0.0230 (19)
C3	0.063 (3)	0.100 (3)	0.106 (4)	0.043 (2)	0.035 (3)	0.037 (3)
C4	0.086 (3)	0.123 (4)	0.105 (4)	0.077 (3)	0.030 (3)	0.040 (3)
C5	0.101 (4)	0.097 (3)	0.103 (4)	0.074 (3)	0.027 (3)	0.042 (3)
C6	0.070 (2)	0.063 (2)	0.074 (3)	0.0389 (19)	0.012 (2)	0.0246 (19)
C7	0.0464 (16)	0.0417 (14)	0.0327 (15)	0.0168 (12)	0.0003 (12)	0.0048 (11)
C8	0.095 (3)	0.085 (3)	0.062 (3)	0.062 (3)	−0.027 (2)	−0.026 (2)
C9	0.139 (5)	0.099 (3)	0.066 (3)	0.076 (4)	−0.047 (3)	−0.042 (3)
C10	0.085 (3)	0.067 (2)	0.049 (2)	0.025 (2)	−0.025 (2)	−0.0065 (18)
C11	0.056 (2)	0.072 (2)	0.057 (2)	0.0264 (18)	−0.0150 (17)	0.0070 (18)
C12	0.0517 (19)	0.0537 (18)	0.051 (2)	0.0236 (15)	−0.0061 (15)	−0.0009 (14)
C13	0.0458 (18)	0.069 (2)	0.051 (2)	0.0222 (16)	0.0090 (15)	0.0204 (16)
C14	0.065 (3)	0.071 (3)	0.065 (3)	0.002 (2)	0.000 (2)	0.017 (2)
C15	0.072 (3)	0.072 (3)	0.103 (4)	0.007 (2)	0.007 (3)	0.027 (3)
C16	0.094 (4)	0.088 (3)	0.114 (5)	0.037 (3)	0.041 (3)	0.055 (3)
C17	0.143 (6)	0.111 (4)	0.074 (4)	0.045 (4)	0.048 (4)	0.044 (3)
C18	0.118 (4)	0.079 (3)	0.057 (3)	0.032 (3)	0.027 (3)	0.019 (2)
C19	0.061 (2)	0.075 (2)	0.0450 (19)	0.0366 (19)	0.0041 (16)	0.0087 (16)
C20	0.073 (3)	0.102 (4)	0.139 (6)	0.037 (3)	−0.008 (3)	0.025 (4)
C21	0.076 (4)	0.144 (6)	0.141 (6)	0.053 (4)	−0.022 (4)	0.008 (5)
C22	0.100 (4)	0.131 (5)	0.098 (4)	0.076 (4)	0.000 (3)	0.036 (4)
C23A	0.115 (6)	0.101 (6)	0.149 (8)	0.046 (5)	−0.024 (5)	0.042 (6)
C24A	0.074 (4)	0.097 (5)	0.123 (7)	0.030 (4)	−0.010 (4)	0.056 (5)
C23B	0.075 (8)	0.108 (10)	0.071 (9)	0.042 (7)	0.003 (6)	0.039 (7)
C24B	0.072 (8)	0.120 (11)	0.079 (9)	0.044 (7)	0.010 (7)	0.025 (8)
C25	0.067 (2)	0.081 (2)	0.0344 (17)	0.040 (2)	0.0002 (15)	0.0023 (16)
C26	0.160 (6)	0.098 (4)	0.068 (3)	0.063 (4)	−0.028 (4)	0.013 (3)
C27	0.262 (11)	0.173 (7)	0.070 (4)	0.132 (8)	−0.045 (6)	0.018 (5)
C28	0.169 (7)	0.193 (8)	0.049 (3)	0.118 (6)	−0.033 (4)	−0.011 (4)
C29	0.110 (4)	0.132 (5)	0.057 (3)	0.051 (4)	−0.025 (3)	−0.022 (3)
C30	0.084 (3)	0.090 (3)	0.050 (2)	0.033 (2)	−0.012 (2)	0.001 (2)
C31	0.074 (3)	0.065 (2)	0.052 (2)	0.039 (2)	−0.0100 (18)	−0.0012 (17)
C32	0.109 (5)	0.112 (4)	0.146 (6)	0.078 (4)	0.022 (4)	0.024 (4)
C33	0.156 (8)	0.155 (7)	0.192 (10)	0.128 (7)	0.005 (7)	0.011 (7)
C34A	0.18 (2)	0.097 (10)	0.099 (11)	0.098 (13)	−0.006 (12)	0.008 (7)
C35A	0.110 (18)	0.078 (7)	0.086 (6)	0.053 (9)	−0.011 (10)	0.022 (5)
C34B	0.08 (2)	0.11 (2)	0.08 (2)	0.064 (15)	−0.004 (14)	0.034 (16)
C35B	0.063 (16)	0.073 (13)	0.076 (13)	0.036 (12)	−0.015 (12)	−0.012 (11)
C36	0.110 (4)	0.065 (2)	0.064 (3)	0.043 (3)	−0.011 (3)	0.003 (2)
C37	0.0583 (19)	0.0487 (16)	0.0424 (17)	0.0267 (15)	0.0042 (14)	0.0050 (13)
C38	0.059 (2)	0.066 (2)	0.115 (4)	0.030 (2)	0.027 (3)	0.013 (2)
C39	0.072 (3)	0.110 (4)	0.141 (6)	0.051 (3)	0.032 (3)	0.012 (4)
C40	0.102 (4)	0.100 (4)	0.094 (4)	0.073 (3)	0.024 (3)	0.004 (3)
C41	0.109 (4)	0.070 (2)	0.063 (3)	0.059 (3)	0.010 (2)	0.0019 (19)
C42	0.078 (3)	0.0532 (19)	0.053 (2)	0.0322 (18)	0.0022 (18)	0.0003 (15)
C43	0.0452 (16)	0.0393 (14)	0.0475 (18)	0.0158 (13)	−0.0006 (13)	0.0087 (12)
C44	0.059 (2)	0.061 (2)	0.055 (2)	0.0025 (17)	−0.0084 (18)	0.0166 (17)
C45	0.055 (2)	0.063 (2)	0.084 (3)	−0.0038 (18)	−0.011 (2)	0.020 (2)
C46	0.050 (2)	0.0533 (19)	0.088 (3)	0.0124 (16)	0.016 (2)	0.028 (2)
C47	0.065 (2)	0.061 (2)	0.058 (2)	0.0255 (18)	0.0190 (19)	0.0211 (17)
C48	0.0528 (19)	0.0471 (16)	0.0490 (19)	0.0187 (14)	0.0042 (15)	0.0081 (14)
N1	0.0472 (14)	0.0375 (12)	0.0385 (13)	0.0175 (11)	−0.0024 (11)	0.0047 (10)
N2	0.0543 (17)	0.0666 (18)	0.0423 (16)	0.0235 (14)	0.0109 (13)	0.0018 (13)
P1	0.0385 (4)	0.0349 (3)	0.0330 (4)	0.0137 (3)	0.0002 (3)	0.0037 (3)
P2	0.0481 (4)	0.0570 (5)	0.0376 (4)	0.0233 (4)	0.0071 (3)	0.0093 (3)
P3	0.0529 (5)	0.0554 (5)	0.0359 (4)	0.0266 (4)	−0.0018 (3)	0.0030 (3)
P4	0.0409 (4)	0.0348 (3)	0.0366 (4)	0.0134 (3)	−0.0001 (3)	0.0051 (3)
S1	0.0479 (4)	0.0441 (4)	0.0349 (4)	0.0051 (3)	0.0033 (3)	0.0037 (3)
S2	0.0552 (5)	0.0414 (4)	0.0464 (4)	0.0202 (3)	0.0145 (4)	0.0108 (3)
S3	0.0453 (4)	0.0600 (4)	0.0358 (4)	0.0225 (4)	−0.0016 (3)	0.0076 (3)
S4	0.0466 (4)	0.0460 (4)	0.0425 (4)	0.0090 (3)	−0.0066 (3)	0.0132 (3)
Pt1	0.04044 (7)	0.03858 (6)	0.03214 (7)	0.01761 (5)	0.00324 (4)	0.00568 (4)

### Geometric parameters (Å, °)

**Table:**

C1—C6	1.386 (5)	C27—H27	0.9300
C1—C2	1.388 (5)	C28—C29	1.359 (10)
C1—P1	1.800 (3)	C28—H28	0.9300
C2—C3	1.381 (6)	C29—C30	1.384 (7)
C2—H2	0.9300	C29—H29	0.9300
C3—C4	1.372 (7)	C30—H30	0.9300
C3—H3	0.9300	C31—C36	1.362 (7)
C4—C5	1.365 (7)	C31—C32	1.377 (7)
C4—H4	0.9300	C31—P3	1.803 (4)
C5—C6	1.375 (6)	C32—C33	1.421 (9)
C5—H5	0.9300	C32—H32	0.9300
C6—H6	0.9300	C33—C34A	1.19 (4)
C7—C12	1.373 (5)	C33—C34B	1.72 (6)
C7—C8	1.378 (5)	C33—H33	0.9300
C7—P1	1.800 (3)	C34A—C35A	1.35 (2)
C8—C9	1.384 (6)	C34A—H34A	0.9300
C8—H8	0.9300	C35A—C36	1.432 (18)
C9—C10	1.360 (7)	C35A—H35A	0.9300
C9—H9	0.9300	C34B—C35B	1.37 (4)
C10—C11	1.355 (6)	C34B—H34B	0.9300
C10—H10	0.9300	C35B—C36	1.35 (4)
C11—C12	1.384 (5)	C35B—H35B	0.9300
C11—H11	0.9300	C36—H36	0.9300
C12—H12	0.9300	C37—C38	1.379 (6)
C13—C18	1.376 (6)	C37—C42	1.393 (5)
C13—C14	1.388 (6)	C37—P4	1.809 (3)
C13—P2	1.802 (4)	C38—C39	1.385 (6)
C14—C15	1.378 (6)	C38—H38	0.9300
C14—H14	0.9300	C39—C40	1.363 (7)
C15—C16	1.361 (8)	C39—H39	0.9300
C15—H15	0.9300	C40—C41	1.349 (7)
C16—C17	1.340 (8)	C40—H40	0.9300
C16—H16	0.9300	C41—C42	1.375 (6)
C17—C18	1.397 (7)	C41—H41	0.9300
C17—H17	0.9300	C42—H42	0.9300
C18—H18	0.9300	C43—C48	1.381 (5)
C19—C24A	1.319 (8)	C43—C44	1.386 (5)
C19—C24B	1.32 (2)	C43—P4	1.808 (3)
C19—C20	1.422 (7)	C44—C45	1.384 (5)
C19—P2	1.817 (4)	C44—H44	0.9300
C20—C21	1.412 (8)	C45—C46	1.375 (6)
C20—H20	0.9300	C45—H45	0.9300
C21—C22	1.359 (9)	C46—C47	1.365 (6)
C21—H21	0.9300	C46—H46	0.9300
C22—C23A	1.312 (11)	C47—C48	1.384 (5)
C22—C23B	1.33 (2)	C47—H47	0.9300
C22—H22	0.9300	C48—H48	0.9300
C23A—C24A	1.395 (12)	N1—P1	1.588 (2)
C23A—H23A	0.9300	N1—P4	1.593 (3)
C24A—H24A	0.9300	N2—P3	1.579 (3)
C23B—C24B	1.39 (3)	N2—P2	1.586 (3)
C23B—H23B	0.9300	P1—S1	2.0278 (11)
C24B—H24B	0.9300	P2—S2	2.0333 (12)
C25—C30	1.369 (6)	P3—S3	2.0325 (12)
C25—C26	1.396 (6)	P4—S4	2.0304 (11)
C25—P3	1.801 (4)	S1—Pt1	2.3286 (8)
C26—C27	1.381 (8)	S2—Pt1	2.3209 (8)
C26—H26	0.9300	S3—Pt1	2.3299 (8)
C27—C28	1.352 (11)	S4—Pt1	2.3339 (8)
			
C6—C1—C2	118.6 (3)	C29—C30—H30	119.6
C6—C1—P1	121.4 (3)	C36—C31—C32	119.5 (5)
C2—C1—P1	119.9 (3)	C36—C31—P3	122.9 (4)
C3—C2—C1	120.6 (4)	C32—C31—P3	117.5 (4)
C3—C2—H2	119.7	C31—C32—C33	117.8 (7)
C1—C2—H2	119.7	C31—C32—H32	121.1
C4—C3—C2	119.6 (4)	C33—C32—H32	121.1
C4—C3—H3	120.2	C34A—C33—C32	125.2 (15)
C2—C3—H3	120.2	C32—C33—C34B	114.4 (18)
C5—C4—C3	120.5 (4)	C34A—C33—H33	117.4
C5—C4—H4	119.8	C32—C33—H33	117.4
C3—C4—H4	119.8	C34B—C33—H33	128.1
C4—C5—C6	120.3 (4)	C33—C34A—C35A	118.6 (15)
C4—C5—H5	119.9	C33—C34A—H34A	120.7
C6—C5—H5	119.9	C35A—C34A—H34A	120.7
C5—C6—C1	120.4 (4)	C34A—C35A—C36	123.2 (15)
C5—C6—H6	119.8	C34A—C35A—H35A	118.4
C1—C6—H6	119.8	C36—C35A—H35A	118.4
C12—C7—C8	118.2 (3)	C35B—C34B—C33	122 (3)
C12—C7—P1	118.8 (2)	C35B—C34B—H34B	119.2
C8—C7—P1	122.9 (3)	C33—C34B—H34B	119.2
C7—C8—C9	120.2 (4)	C36—C35B—C34B	107 (4)
C7—C8—H8	119.9	C36—C35B—H35B	126.6
C9—C8—H8	119.9	C34B—C35B—H35B	126.6
C10—C9—C8	120.8 (4)	C35B—C36—C31	137 (2)
C10—C9—H9	119.6	C31—C36—C35A	115.4 (16)
C8—C9—H9	119.6	C35B—C36—H36	100.0
C11—C10—C9	119.4 (4)	C31—C36—H36	122.3
C11—C10—H10	120.3	C35A—C36—H36	122.3
C9—C10—H10	120.3	C38—C37—C42	117.9 (4)
C10—C11—C12	120.5 (4)	C38—C37—P4	120.2 (3)
C10—C11—H11	119.8	C42—C37—P4	121.7 (3)
C12—C11—H11	119.8	C37—C38—C39	120.3 (4)
C7—C12—C11	120.9 (3)	C37—C38—H38	119.9
C7—C12—H12	119.6	C39—C38—H38	119.9
C11—C12—H12	119.6	C40—C39—C38	120.8 (5)
C18—C13—C14	117.8 (4)	C40—C39—H39	119.6
C18—C13—P2	120.9 (3)	C38—C39—H39	119.6
C14—C13—P2	121.3 (3)	C41—C40—C39	119.4 (4)
C15—C14—C13	121.9 (5)	C41—C40—H40	120.3
C15—C14—H14	119.0	C39—C40—H40	120.3
C13—C14—H14	119.0	C40—C41—C42	121.2 (4)
C16—C15—C14	118.5 (5)	C40—C41—H41	119.4
C16—C15—H15	120.7	C42—C41—H41	119.4
C14—C15—H15	120.7	C41—C42—C37	120.4 (4)
C17—C16—C15	121.4 (5)	C41—C42—H42	119.8
C17—C16—H16	119.3	C37—C42—H42	119.8
C15—C16—H16	119.3	C48—C43—C44	119.3 (3)
C16—C17—C18	120.4 (5)	C48—C43—P4	119.2 (2)
C16—C17—H17	119.8	C44—C43—P4	121.5 (3)
C18—C17—H17	119.8	C45—C44—C43	120.2 (4)
C13—C18—C17	119.8 (5)	C45—C44—H44	119.9
C13—C18—H18	120.1	C43—C44—H44	119.9
C17—C18—H18	120.1	C46—C45—C44	120.0 (4)
C24A—C19—C24B	49.2 (10)	C46—C45—H45	120.0
C24A—C19—C20	117.7 (5)	C44—C45—H45	120.0
C24B—C19—C20	100.7 (10)	C47—C46—C45	119.9 (3)
C24A—C19—P2	121.2 (4)	C47—C46—H46	120.1
C24B—C19—P2	121.7 (9)	C45—C46—H46	120.1
C20—C19—P2	120.7 (4)	C46—C47—C48	120.8 (4)
C21—C20—C19	118.1 (6)	C46—C47—H47	119.6
C21—C20—H20	120.9	C48—C47—H47	119.6
C19—C20—H20	120.9	C43—C48—C47	119.8 (3)
C22—C21—C20	120.4 (6)	C43—C48—H48	120.1
C22—C21—H21	119.8	C47—C48—H48	120.1
C20—C21—H21	119.8	P1—N1—P4	125.54 (17)
C23A—C22—C23B	47.3 (10)	P3—N2—P2	131.51 (19)
C23A—C22—C21	119.9 (6)	N1—P1—C1	112.15 (15)
C23B—C22—C21	102.8 (10)	N1—P1—C7	106.37 (14)
C23A—C22—H22	120.1	C1—P1—C7	107.27 (15)
C23B—C22—H22	117.1	N1—P1—S1	116.37 (11)
C21—C22—H22	120.1	C1—P1—S1	110.13 (11)
C22—C23A—C24A	120.2 (8)	C7—P1—S1	103.75 (10)
C22—C23A—H23A	119.9	N2—P2—C13	108.81 (18)
C24A—C23A—H23A	119.9	N2—P2—C19	110.03 (18)
C19—C24A—C23A	122.5 (8)	C13—P2—C19	105.46 (18)
C19—C24A—H24A	118.7	N2—P2—S2	116.77 (12)
C23A—C24A—H24A	118.7	C13—P2—S2	103.90 (13)
C22—C23B—C24B	124.3 (16)	C19—P2—S2	111.06 (13)
C22—C23B—H23B	117.8	N2—P3—C25	107.48 (18)
C24B—C23B—H23B	117.8	N2—P3—C31	110.2 (2)
C19—C24B—C23B	117.9 (17)	C25—P3—C31	105.70 (19)
C19—C24B—H24B	121.0	N2—P3—S3	117.98 (12)
C23B—C24B—H24B	121.0	C25—P3—S3	103.84 (13)
C30—C25—C26	118.9 (4)	C31—P3—S3	110.71 (15)
C30—C25—P3	121.8 (3)	N1—P4—C43	107.67 (15)
C26—C25—P3	119.1 (4)	N1—P4—C37	107.40 (15)
C27—C26—C25	119.0 (6)	C43—P4—C37	105.66 (15)
C27—C26—H26	120.5	N1—P4—S4	117.64 (10)
C25—C26—H26	120.5	C43—P4—S4	105.99 (11)
C28—C27—C26	121.3 (7)	C37—P4—S4	111.77 (12)
C28—C27—H27	119.3	P1—S1—Pt1	109.16 (4)
C26—C27—H27	119.3	P2—S2—Pt1	104.97 (4)
C27—C28—C29	120.2 (6)	P3—S3—Pt1	104.36 (4)
C27—C28—H28	119.9	P4—S4—Pt1	110.20 (4)
C29—C28—H28	119.9	S2—Pt1—S1	82.41 (3)
C28—C29—C30	119.8 (6)	S2—Pt1—S3	90.87 (3)
C28—C29—H29	120.1	S1—Pt1—S3	172.36 (3)
C30—C29—H29	120.1	S2—Pt1—S4	171.73 (3)
C25—C30—C29	120.8 (5)	S1—Pt1—S4	101.46 (3)
C25—C30—H30	119.6	S3—Pt1—S4	84.75 (3)
			
C6—C1—C2—C3	−1.5 (6)	P4—N1—P1—C1	−76.6 (2)
P1—C1—C2—C3	−179.4 (4)	P4—N1—P1—C7	166.4 (2)
C1—C2—C3—C4	1.7 (8)	P4—N1—P1—S1	51.4 (2)
C2—C3—C4—C5	−0.8 (9)	C6—C1—P1—N1	160.7 (3)
C3—C4—C5—C6	−0.3 (9)	C2—C1—P1—N1	−21.5 (3)
C4—C5—C6—C1	0.6 (8)	C6—C1—P1—C7	−82.9 (3)
C2—C1—C6—C5	0.3 (7)	C2—C1—P1—C7	94.9 (3)
P1—C1—C6—C5	178.2 (4)	C6—C1—P1—S1	29.4 (3)
C12—C7—C8—C9	−1.2 (7)	C2—C1—P1—S1	−152.8 (3)
P1—C7—C8—C9	175.6 (4)	C12—C7—P1—N1	−44.6 (3)
C7—C8—C9—C10	0.5 (9)	C8—C7—P1—N1	138.6 (4)
C8—C9—C10—C11	0.7 (9)	C12—C7—P1—C1	−164.8 (3)
C9—C10—C11—C12	−1.3 (7)	C8—C7—P1—C1	18.4 (4)
C8—C7—C12—C11	0.6 (6)	C12—C7—P1—S1	78.6 (3)
P1—C7—C12—C11	−176.3 (3)	C8—C7—P1—S1	−98.1 (4)
C10—C11—C12—C7	0.6 (6)	P3—N2—P2—C13	147.7 (3)
C18—C13—C14—C15	1.5 (7)	P3—N2—P2—C19	−97.2 (3)
P2—C13—C14—C15	−178.8 (4)	P3—N2—P2—S2	30.5 (3)
C13—C14—C15—C16	0.2 (8)	C18—C13—P2—N2	11.3 (4)
C14—C15—C16—C17	−1.9 (10)	C14—C13—P2—N2	−168.3 (4)
C15—C16—C17—C18	1.7 (11)	C18—C13—P2—C19	−106.7 (4)
C14—C13—C18—C17	−1.7 (8)	C14—C13—P2—C19	73.7 (4)
P2—C13—C18—C17	178.7 (5)	C18—C13—P2—S2	136.4 (4)
C16—C17—C18—C13	0.1 (10)	C14—C13—P2—S2	−43.2 (4)
C24A—C19—C20—C21	3.5 (10)	C24A—C19—P2—N2	52.1 (7)
C24B—C19—C20—C21	−45.8 (13)	C24B—C19—P2—N2	110.5 (14)
P2—C19—C20—C21	177.0 (5)	C20—C19—P2—N2	−121.1 (4)
C19—C20—C21—C22	−0.8 (11)	C24A—C19—P2—C13	169.3 (7)
C20—C21—C22—C23A	−7.6 (14)	C24B—C19—P2—C13	−132.3 (14)
C20—C21—C22—C23B	40.2 (14)	C20—C19—P2—C13	−3.9 (5)
C23B—C22—C23A—C24A	−66.5 (14)	C24A—C19—P2—S2	−78.7 (7)
C21—C22—C23A—C24A	13.0 (17)	C24B—C19—P2—S2	−20.3 (14)
C24B—C19—C24A—C23A	81.6 (15)	C20—C19—P2—S2	108.1 (4)
C20—C19—C24A—C23A	1.8 (14)	P2—N2—P3—C25	−147.3 (3)
P2—C19—C24A—C23A	−171.7 (9)	P2—N2—P3—C31	98.0 (3)
C22—C23A—C24A—C19	−10.3 (18)	P2—N2—P3—S3	−30.5 (3)
C23A—C22—C23B—C24B	84 (2)	C30—C25—P3—N2	−145.9 (4)
C21—C22—C23B—C24B	−35 (3)	C26—C25—P3—N2	39.5 (5)
C24A—C19—C24B—C23B	−65 (2)	C30—C25—P3—C31	−28.3 (4)
C20—C19—C24B—C23B	52 (2)	C26—C25—P3—C31	157.1 (4)
P2—C19—C24B—C23B	−171.0 (15)	C30—C25—P3—S3	88.3 (4)
C22—C23B—C24B—C19	−14 (3)	C26—C25—P3—S3	−86.3 (4)
C30—C25—C26—C27	0.3 (10)	C36—C31—P3—N2	−146.5 (4)
P3—C25—C26—C27	175.1 (6)	C32—C31—P3—N2	35.7 (5)
C25—C26—C27—C28	−0.5 (13)	C36—C31—P3—C25	97.6 (4)
C26—C27—C28—C29	−0.4 (14)	C32—C31—P3—C25	−80.2 (5)
C27—C28—C29—C30	1.5 (12)	C36—C31—P3—S3	−14.2 (4)
C26—C25—C30—C29	0.8 (8)	C32—C31—P3—S3	168.0 (4)
P3—C25—C30—C29	−173.8 (4)	P1—N1—P4—C43	128.1 (2)
C28—C29—C30—C25	−1.8 (9)	P1—N1—P4—C37	−118.5 (2)
C36—C31—C32—C33	−0.2 (9)	P1—N1—P4—S4	8.5 (2)
P3—C31—C32—C33	177.7 (6)	C48—C43—P4—N1	22.8 (3)
C31—C32—C33—C34A	−0.1 (17)	C44—C43—P4—N1	−159.5 (3)
C31—C32—C33—C34B	3(2)	C48—C43—P4—C37	−91.7 (3)
C32—C33—C34A—C35A	4(3)	C44—C43—P4—C37	85.9 (3)
C34B—C33—C34A—C35A	−12 (10)	C48—C43—P4—S4	149.5 (2)
C33—C34A—C35A—C36	−7(3)	C44—C43—P4—S4	−32.8 (3)
C34A—C33—C34B—C35B	152 (15)	C38—C37—P4—N1	37.4 (4)
C32—C33—C34B—C35B	−14 (5)	C42—C37—P4—N1	−138.9 (3)
C33—C34B—C35B—C36	18 (6)	C38—C37—P4—C43	152.1 (4)
C34B—C35B—C36—C31	−18 (5)	C42—C37—P4—C43	−24.1 (3)
C34B—C35B—C36—C35A	9(4)	C38—C37—P4—S4	−93.0 (4)
C32—C31—C36—C35B	9(3)	C42—C37—P4—S4	90.7 (3)
P3—C31—C36—C35B	−169 (3)	N1—P1—S1—Pt1	−57.10 (12)
C32—C31—C36—C35A	−2.6 (11)	C1—P1—S1—Pt1	71.94 (12)
P3—C31—C36—C35A	179.7 (9)	C7—P1—S1—Pt1	−173.53 (11)
C34A—C35A—C36—C35B	−154 (6)	N2—P2—S2—Pt1	−52.29 (15)
C34A—C35A—C36—C31	6(2)	C13—P2—S2—Pt1	−172.08 (13)
C42—C37—C38—C39	−0.4 (7)	C19—P2—S2—Pt1	74.98 (15)
P4—C37—C38—C39	−176.8 (5)	N2—P3—S3—Pt1	51.52 (15)
C37—C38—C39—C40	−1.4 (10)	C25—P3—S3—Pt1	170.28 (14)
C38—C39—C40—C41	2.6 (10)	C31—P3—S3—Pt1	−76.69 (14)
C39—C40—C41—C42	−2.0 (9)	N1—P4—S4—Pt1	−51.14 (13)
C40—C41—C42—C37	0.2 (7)	C43—P4—S4—Pt1	−171.57 (11)
C38—C37—C42—C41	0.9 (6)	C37—P4—S4—Pt1	73.80 (13)
P4—C37—C42—C41	177.3 (3)	P2—S2—Pt1—S1	−118.08 (5)
C48—C43—C44—C45	−1.2 (6)	P2—S2—Pt1—S3	65.56 (5)
P4—C43—C44—C45	−178.9 (4)	P1—S1—Pt1—S2	−161.21 (5)
C43—C44—C45—C46	−0.1 (7)	P1—S1—Pt1—S4	11.37 (5)
C44—C45—C46—C47	1.2 (7)	P3—S3—Pt1—S2	−64.69 (5)
C45—C46—C47—C48	−1.1 (7)	P3—S3—Pt1—S4	122.34 (5)
C44—C43—C48—C47	1.4 (5)	P4—S4—Pt1—S1	32.08 (5)
P4—C43—C48—C47	179.1 (3)	P4—S4—Pt1—S3	−152.41 (5)
C46—C47—C48—C43	−0.2 (6)		
